# Supplementary material for: The Lipid Receptor G2A (GPR132) Mediates Macrophage Migration in Nerve Injury-Induced Neuropathic Pain
Source: Cells. 2020 Jul 21;9(7):1740. doi: 10.3390/cells9071740 (PMC7409160; doi:10.3390/cells9071740)
Supplement: Supplementary file 1 [file cells-09-01740-s001.pdf]

Supplementary Information

The Lipid Receptor G2A (GPR132) Mediates Macrophage Migration in Nerve Injury-Induced Neuropathic Pain

Tabea Osthues [1], Béla Zimmer [2], Vittoria Rimola [2], Kevin Klann [3], Karin Schilling [2], Praveen Mathoor [4], Carlo Angioni [2], Andreas Weigert [4], Gerd Geisslinger [1,2], Christian Münch [5], Klaus Scholich [1,2] and Marco Sisignano [1,2,*]

[1] Fraunhofer Institute for Molecular Biology and Applied Ecology IME, Branch for Translational Medicine and Pharmacology TMP, Theodor-Stern-Kai 7, 60596 Frankfurt am Main, Germany; Tabea.Osthues@ime.fraunhofer.de (T.O.); geisslinger@em.uni-frankfurt.de (G.G.); scholich@em.uni-frankfurt.de (K.S.)

[2] Institute of Clinical Pharmacology, *pharmazentrum frankfurt/ZAFES*, University Hospital, Goethe-University, D-60590 Frankfurt am Main, Germany; Zimmer@med.uni-frankfurt.de (B.Z.); rimola@med.uni-frankfurt.de (V.R.); Schilling@med.uni-frankfurt.de (K.S.); angioni@em.uni-frankfurt.de (C.A.)

[3] Institute of Biochemistry II, Faculty of Medicine, University Hospital, Goethe-University, D-60590 Frankfurt am Main, Germany; klann@em.uni-frankfurt.de

[4] Institute of Biochemistry I, Faculty of Medicine, Goethe-University, D-60590 Frankfurt am Main, Germany, Frankfurt Cancer Institute, D-60590 Frankfurt am Main, Germany; Cardio-Pulmonary Institute, D-60590 Frankfurt am Main, Germany; mathoor@biochem.uni-frankfurt.de (P.M.); weigert@biochem.uni-frankfurt.de (A.W.)

[5] Institute of Biochemistry II, Faculty of Medicine, University Hospital, Goethe-University, D-60590 Frankfurt am Main, Germany; Frankfurt Cancer Institute, D-60590 Frankfurt am Main, Germany; Cardio-Pulmonary Institute, D-60590 Frankfurt am Main, Germany; ch.muench@em.uni-frankfurt.de

* Correspondence: Marco.Sisignano@med.uni-frankfurt.de; Tel.: +49 69-6301-87819

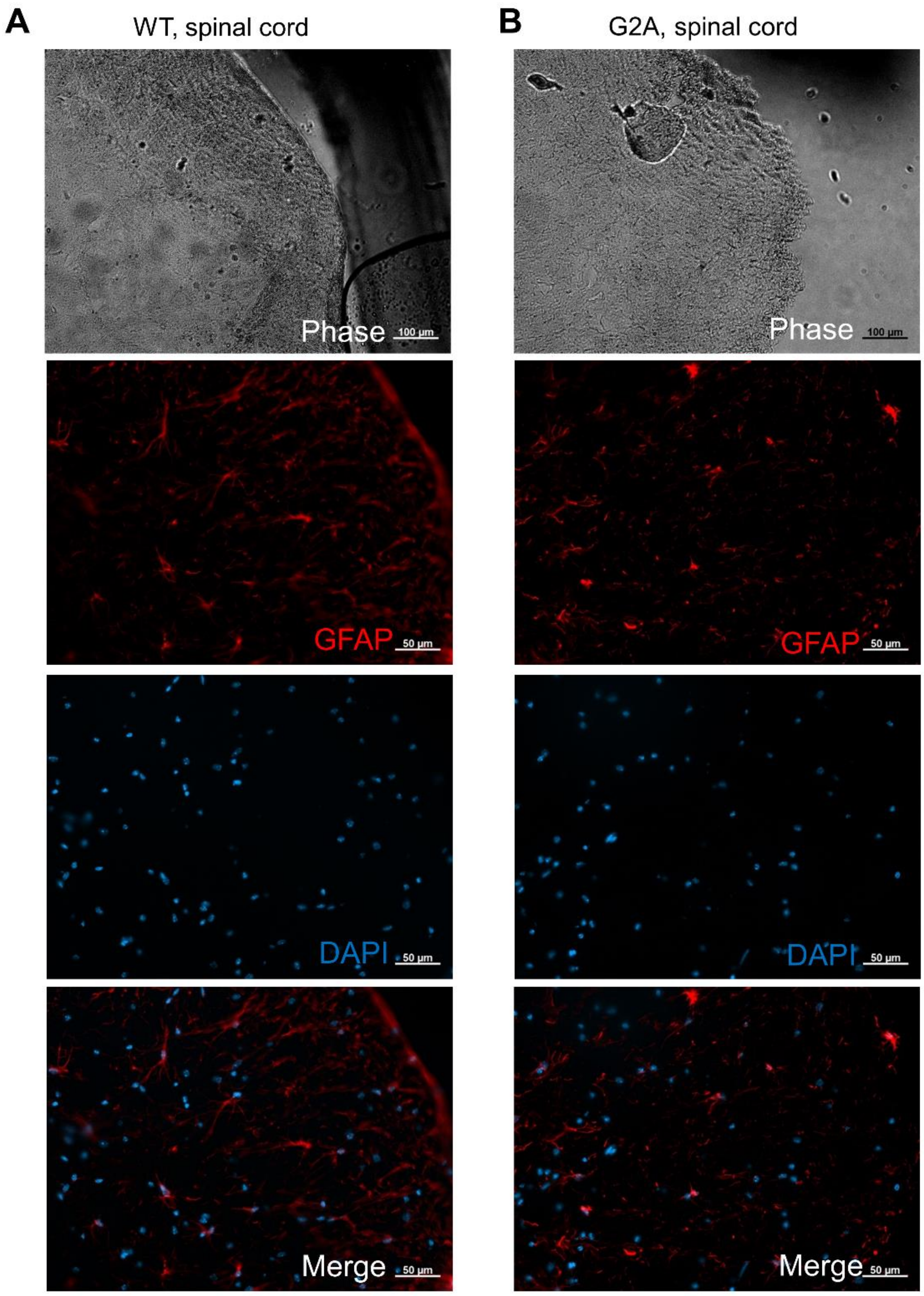

Figure S1. Activated astrocytes in spinal cord 7d after SNI. (**A**) Representative immunohistochemistry images of astrocyte staining (GFAP) of ipsilateral dorsal site of spinal cord in wild-type mice. (**B**) Representative immunohistochemistry images of astrocyte staining (GFAP) of ipsilateral dorsal site of spinal cord in male wild-type mice.

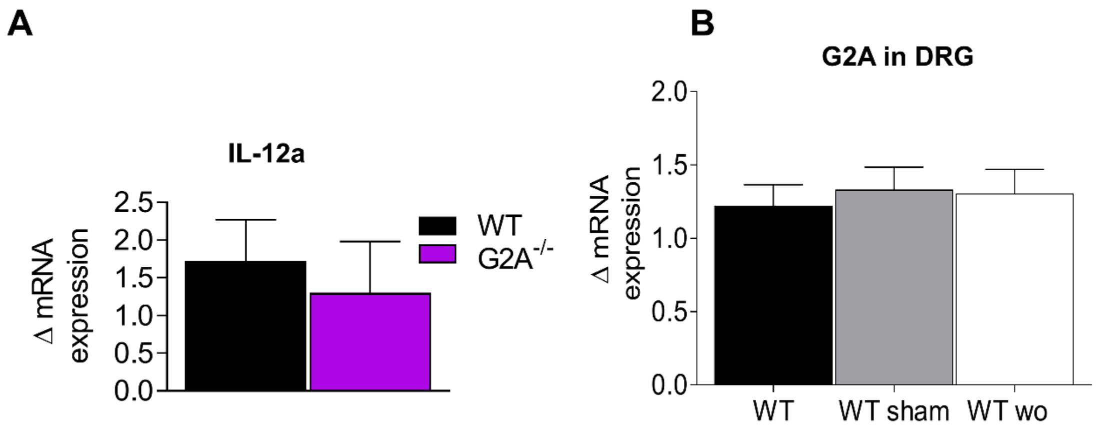

Figure S2. Cytokine and chemokine production in nervous tissue after SNI surgery. (**A**) Relative mRNA expression of IL-12a in DRGs 7d after SNI in WT and G2A$^{-/-}$ mice, n=3 male mice per group. (**B**) Relative mRNA expression of G2A receptor in ipsilateral site of L4 to L6 DRG in wild-type mice (WT) 7 d after SNI, after sham-surgery (WT sham) or without treatment (WT wo). n=8-10 male and female animals per group. WT ipsilateral site is shown in black, WT sham site in grey and WT without any treatment (WT wo) in white. G2A$^{-/-}$ ipsilateral site is depicted in in purple. Data represents mean ± SEM. *p<0.05, one-way ANOVA.

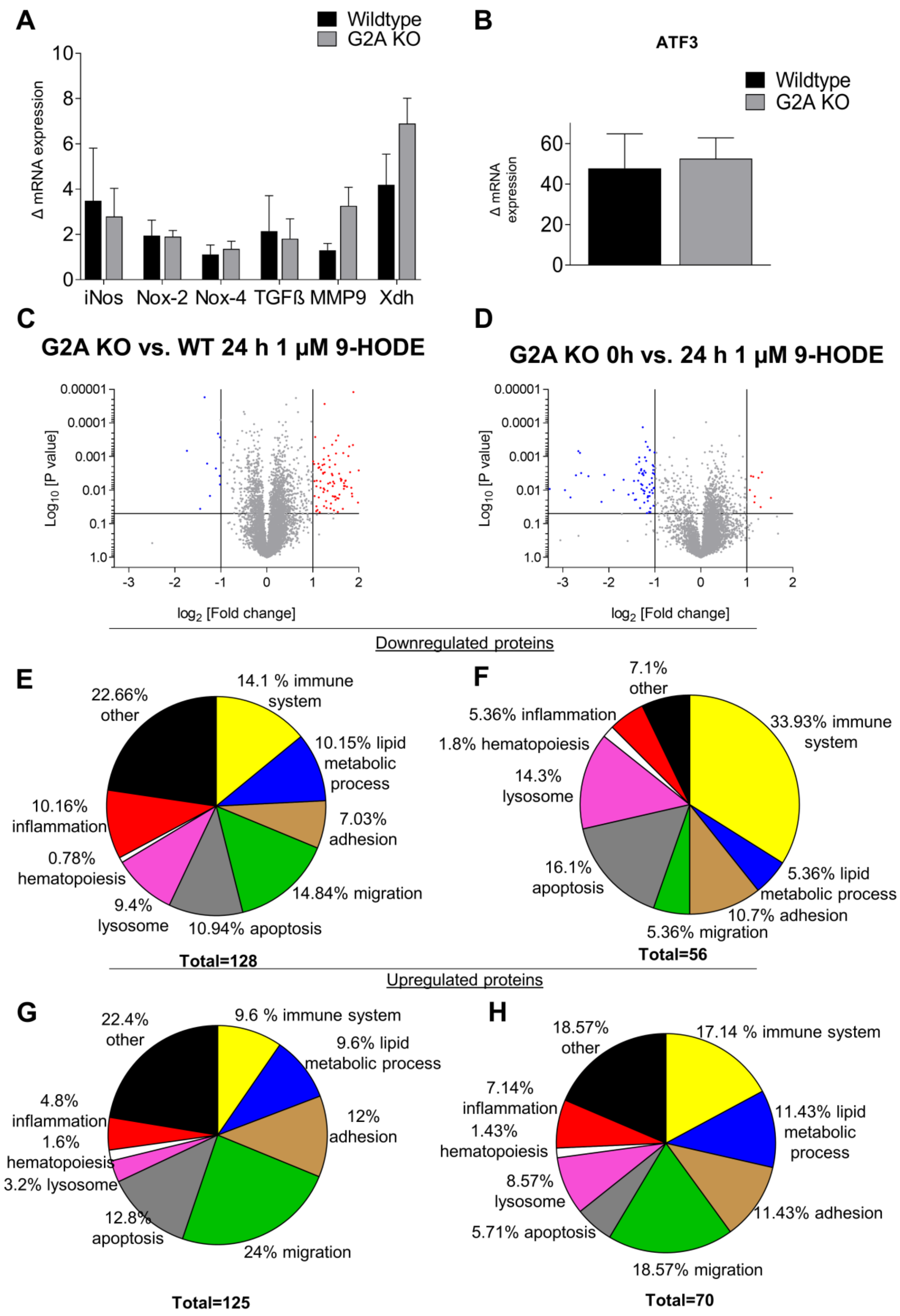

Figure S3. Stress reactions in G2A-deficient mice and in BMDMs after stimulation with 9-HODE. (**A**) Relative mRNA expression of oxidative stress markers in dorsal root ganglia (DRGs) of wild-type (black) and G2A-deficient (G2A-KO, grey) mice 7 d after SNI surgery: inducible nitric oxide synthase (iNOS), NADPH oxidase 2 (NOX-2) and NOX-4, xanthine dehydrogenase (Xdh),

matrixmetalloproteinase 9 (MMP9) and transforming growth factor β (TGFβ). Two-way ANOVA with Bonferroni's post-hoc test. (**B**) Relative mRNA expression of neuronal stress marker activating transcription factor 3 (ATF3) in DRGs of wild-type (black) and G2A-deficient mice (grey) 7 d after SNI surgery. n=4-8 animals per group, male. Data represents mean ± SEM, unpaired t-test. (**C**) Volcano plot of > 6.000 regulated proteins after 24 h stimulation with 1 μM 9-HODE in G2A-deficient BMDMs compared with wild-type mice (WT). (**D**) Volcano plot of > 6.000 regulated proteins in G2A-deficient BMDMs without and after 24 h stimulation with 1 μM 9. Fold change (FC) [log2] is plotted against *P* values [Log10]. Significant downregulated proteins are depicted in blue, red represents upregulated proteins. (**E**) Percentage of downregulated proteins in G2A-deficient BMDMs 24 h after 1 μM 9-HODE stimulation compared to treated WT. (**F**) Percentage of downregulated proteins in G2A-deficient BMDMs 24 h after 1 μM 9-HODE stimulation compared to untreated G2A-deficient BMDMs, clustered in groups. (**G**) Percentage of upregulated proteins in G2A-deficient BMDMs 24 h after 1 μM 9-HODE stimulation compared to treated WT. (**H**) Percentage of upregulated proteins in G2A-deficient BMDMs 24h after 1 μM 9-HODE stimulation compared to untreated G2A-deficient BMDMs, clustered in groups. yellow: immune system, blue: lipid metabolic process, brown: adhesion, green: migration, grey: apoptosis, pink: lysosome, red: inflammation, white: hematopoiesis, black: other.

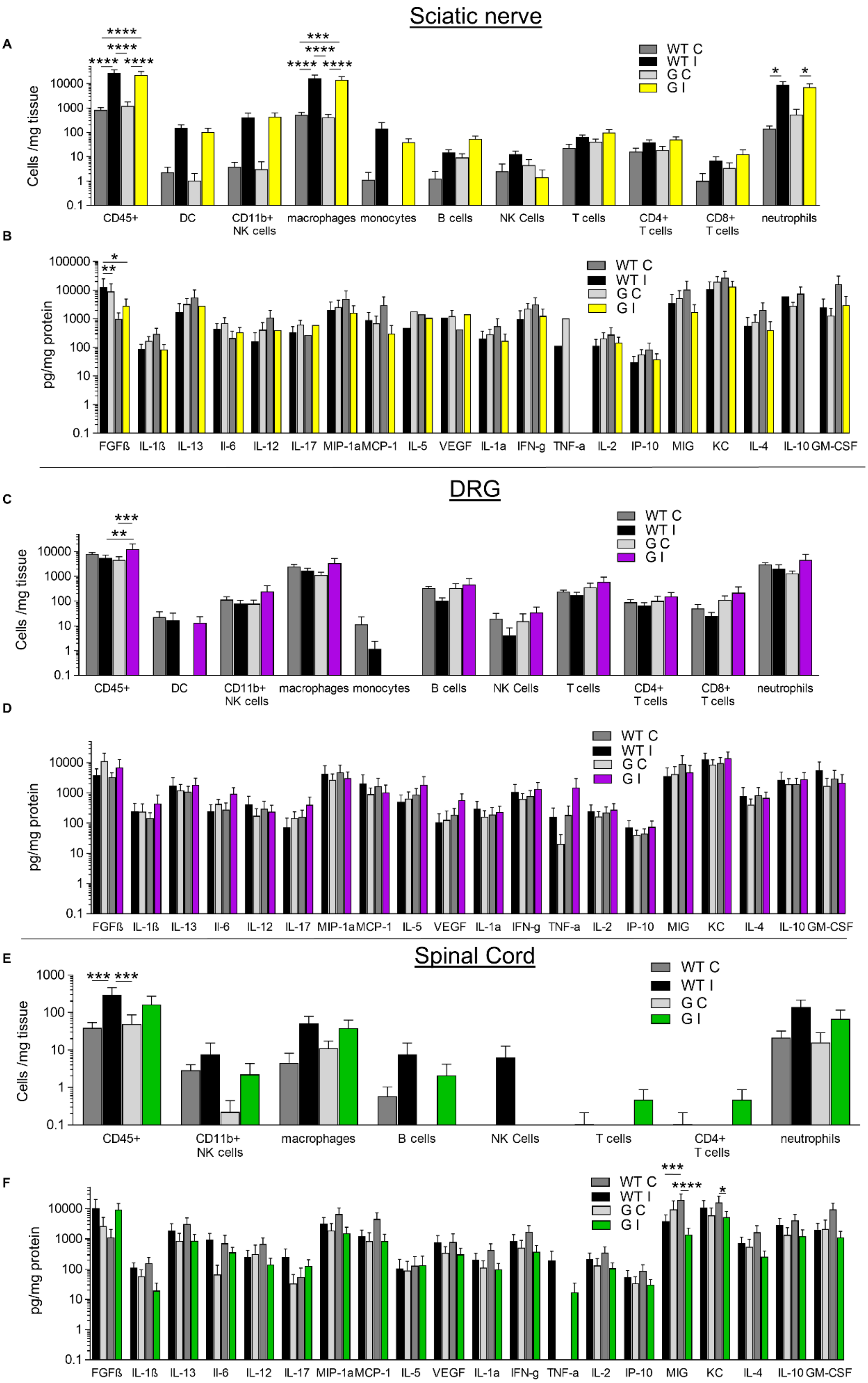

Figure S4. Immune cell number, cytokine and chemokine production in nervous tissue 1d after SNI-treatment. (**A**) Number of immune cells in ipsi- and contralateral site of sciatic nerve (SN) 1 d after SNI surgery in wild-type (WT) and G2A-deficient mice (G2A$^{-/-}$). n=5 animals per group, male and female, Two-way ANOVA with Bonferroni's post-hoc test. (**B**) Concentrations of different

cytokines, chemokines and growth factors in ipsi- and contralateral site of SN 1 d after SNI in wild-type (black) and G2A-deficient mice from n=5 animals per group, male and female, multiple t-test. (**C**) Number of immune cells in ipsi- and contralateral site of L4-L6 dorsal root ganglia (DRG) 1 d after SNI surgery in wild-type and G2A-deficient mice from n=5 animals per group, male and female, Two-way ANOVA with Bonferroni's post-hoc test. (**D**) Concentrations of different cytokines, chemokines and growth factors in ipsi- and contralateral site of L4-L5 DRGs 1 d after SNI surgery in wild-type and G2A-deficient mice from n=5 animals per group, male and female, multiple t-test. (**E**) Number of immune cells in ipsi- and contralateral site of spinal cord 1 d after SNI surgery in wild-type and G2A-deficient mice from n=5 male and female animals per group, Two-way ANOVA with Bonferroni's post-hoc test. (**F**) Concentrations of different cytokines, chemokines and growth factors ipsi- and contralateral site of spinal cord 1 d after SNI surgery in wild-type and G2A-deficient mice. n=5 animals per group, male and female, multiple t-test. Data represents mean ± SEM. Ipsilateral site of WT mice are shown in black. Ipsilateral site in SN is shown in yellow, in DRG in purple and in SC in green. Contralateral site of WT mice are depicted in dark grey, contralateral site of G2A$^{-/-}$ mice is shown in light grey. Abbr.: SEM: standard error of the mean, wo: without treatment, FGF: fibroblast growth factor, IL: interleukin, MIP: macrophage inflammatory protein, MCP: methyl-accepting chemotaxis protein, IFN: interferon, TNF: transforming growth factor, IP: interferon gamma-induced protein, MIG: monokine induced by IFN-gamma, KC: platelet-factor 4-type cytokine, G2A KO: G2A knockout, DRG: dorsal root ganglion, GM-CSF: granulocyte-macrophage colony-stimulating factor, DC: dendritic cells, NK: natural killer.

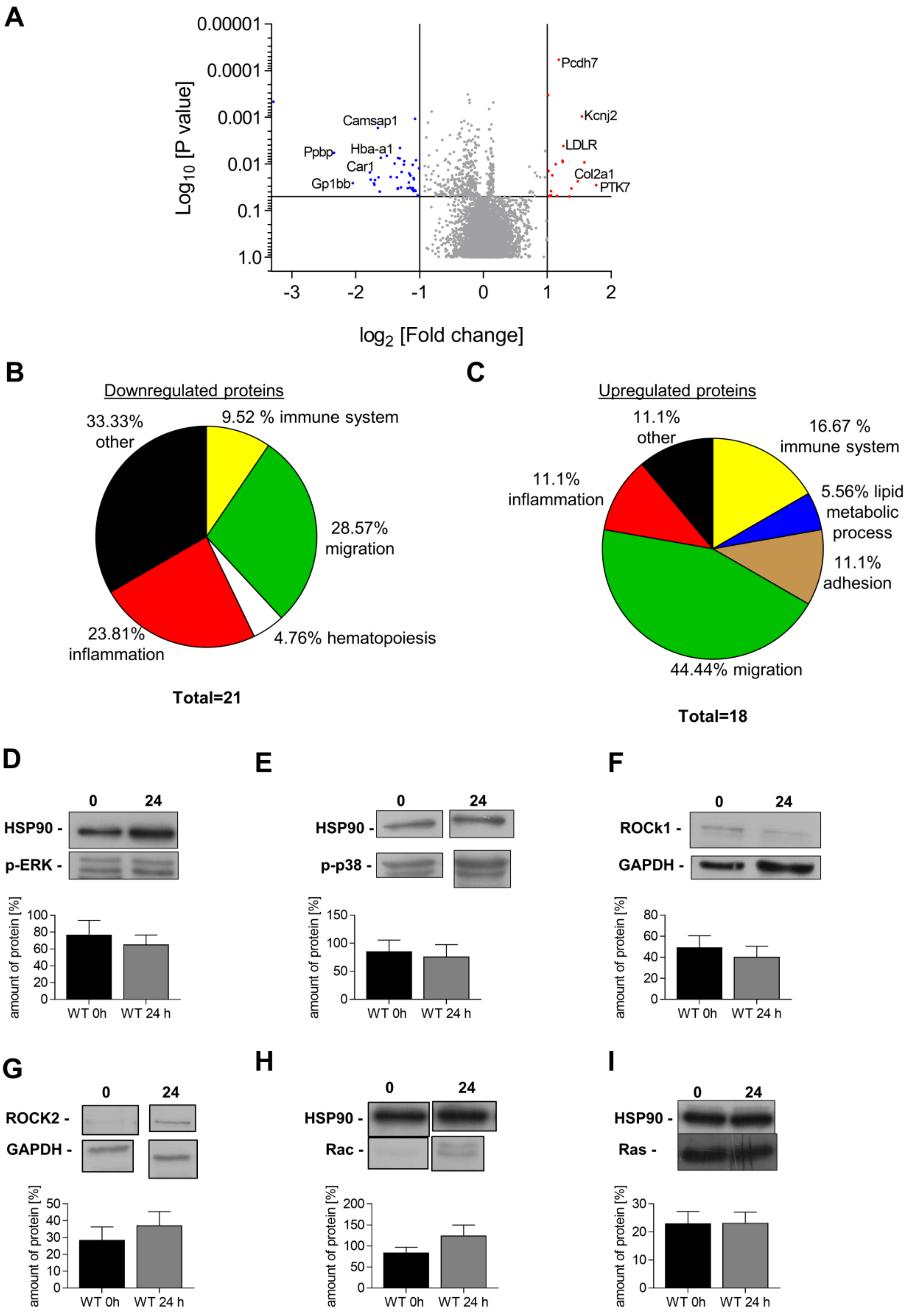

Figure S5. Effect of 9-HODE on migratory signaling pathways. (**A**) Volcano plot of > 6.000 regulated proteins without stimulation in wild-type (WT) BMDMs compared to G2A-deficient (G2A$^{-/-}$) BMDMs. Fold change (FC) [log2] is plotted against *P* values [Log10]. Significant downregulated proteins are depicted in blue, red represents upregulated proteins. Experiments were performed in triplicates,

bone marrow derived macrophages from male mice. (**B**) Percentage of downregulated proteins in untreated WT compared to untreated G2A$^{-/-}$. (**C**) Percentage of upregulated proteins in untreated WT compared to untreated G2A$^{-/-}$, clustered in groups. yellow: immune system, blue: lipid metabolic process, brown: adhesion, green: migration, grey: apoptosis, pink: lysosome, red: inflammation, white: hematopoiesis, black: other. (**D, E**) Representative Western Blots of phosphorylated ERK and p38 in BMDMs stimulated with 1 μM 9-HODE for 0 h (WT 0 h, black) and 24 h (WT 24 h, grey). As endogenous control HSP90 was used. n=5-11. (**F, G**) Representative Western Blots of ROCK1 and ROCK2 in BMDMs stimulated with 1 μM 9-HODE for 0 h (WT 0 h, black) and 24 h (WT 24 h, grey). As endogenous control GAPDH was used. n=5-11 male mice per group. (**H, I**) Representative Western Blots of Rac and Ras in BMDMs stimulated with 1 μM 9-HODE for 0 h (WT 0 h, black) and 24 h (WT 24 h, grey). As endogenous control HSP90 was used. n=8-10 male mice per group. Data represents mean ± SEM, unpaired t-test. Abbr.: SEM: standard error of the mean, HSP: heat shock protein, ROCK: rho-associated protein kinase, Rac: Ras-related C3 botulinum toxin substrate 1, Ras: rat sarcoma, p-38:p-38 mitogen-activated protein kinase , ERK: extracellular signal-regulated kinase.

Supplementary Table 1. Overview of regulated proteins in wild-type BMDMs treated with 1 µM 9-HODE for 24 h. Description according to KEGG pathway database.

Downregulated		Upregulated	
**A) Immune system processes**			
**B2m**	Antigen processing and presentation	**Alcam**	Cell adhesion molecule, hematopoiesis, adaptive immune response
**Blnk**	B cell receptor signaling pathway; Primary immunodeficiency; NF-kappa B signaling pathway; Osteoclast differentiation	**Cd72**	B cell receptor signaling pathway
**C3**	Complement and coagulation cascades; Viral carcinogenesis; Chagas disease (American trypanosomiasis); Systemic lupus erythematosus; Phagosome	**Lilrb4**	Osteoclast differentiation, B cell receptor signaling pathway
**Ccl9**	Cytokine-cytokine receptor interaction; Chemokine signaling pathway	**Lyn**	B cell receptor signaling pathway
**Ctsl**	Apoptosis; Phagosome; Antigen processing and presentation; Lysosome	**Pik3ap1**	B cell receptor signaling pathway, PI3K-Akt signaling pathway
**Fcgr**	Phagocytosis-promoting receptors, B cell receptor signaling, Natural killer cell mediated cytotoxicity; Staphylococcus aureus infection; Systemic lupus erythematosus; Phagosome; Tuberculosis		
**Lgmn**	Lysosome; Antigen processing and presentation		
**Ly96**	NF-kappa B signaling pathway; Toxoplasmosis; Toll-like receptor signaling pathway; Pertussis		
**Notch2**	Notch signaling pathway; Th1 and Th2 cell differentiation		
**Plau**	NF-kappa B signaling pathway; Complement and coagulation cascades		
**Plin2**	PPAR signaling pathway		
**Tlr8**	Toll-like receptor signaling pathway		
B) Lipid metabolic processes			
**Abca1**	ABC transporters; Fat digestion and absorption; Lysosomal membrane protein	**Acadvl**	Metabolic pathways; Fatty acid degradation; Fatty acid metabolism
**Acaa2**	Metabolic pathways; Fatty acid elongation; Fatty acid metabolism; Fatty acid degradation	**Cept1**	Ether lipid metabolism; Glycerophospholipid metabolism
**Acadsb**	Fatty acid degradation; Valine, leucine and isoleucine degradation; Metabolic pathways; Fatty acid metabolism	**Chkb**	Metabolic pathways; Glycerophospholipid metabolism
**Acat1**	Metabolic pathways; Fatty acid metabolism; Fatty acid degradation	**Dgkh, Dgkz**	Glycerophospholipid metabolism
**Acox1**	alpha-Linolenic acid metabolism; Biosynthesis of unsaturated fatty acids; PPAR signaling pathway; Fatty acid degradation; Fatty acid metabolism	**Fads1**	Biosynthesis of unsaturated fatty acids; Fatty acid metabolism
**Aldh1b1**	Lysine degradation; Pyruvate metabolism; Glycolysis / Gluconeogenesis; Glycerolipid metabolism; Fatty acid degradation;	**Fasn**	Fatty acid biosynthesis; Metabolic pathways; Fatty acid metabolism

	Metabolic pathways; glycerolipild metabolism		
**Echs1**	Fatty acid metabolism; Fatty acid elongation; Fatty acid degradation; Metabolic pathways; Carbon metabolism; fatty acid elongation	**Gnpat**	Peroxisome; Glycerophospholipid metabolism
**Gpcpd1**	Glycerophospholipid metabolism	**Lpl**	Glycerolipid metabolism; PPAR signaling pathway
**GPx1**	Arachidonic acid metabolism; Glutathione metabolism	**Hacd3**	Fatty acid metabolism; Biosynthesis of unsaturated fatty acids; Fatty acid elongation
**Hadh**	Fatty acid metabolism; Fatty acid elongation; Fatty acid degradation;	**Smpd2**	Sphingolipid metabolism; Sphingolipid signaling pathway
**Neu1**	Lysosome; Sphingolipid metabolism		
**Pcyt1a**	Glycerophospholipid metabolism;		
**Pld3**	Glycerophospholipid metabolism; Metabolic pathways; Ether lipid metabolism		
**Sdha**	Alzheimer's disease; Huntington's disease; Oxidative phosphorylation; Non-alcoholic fatty liver disease (NAFLD); Metabolic pathways		
**Smpd1**	Sphingolipid signaling pathway; Sphingolipid metabolism; Lysosome		
**Akr1a1**	Metabolic pathways; Glycerolipid metabolism; Glycolysis / Gluconeogenesis		
C) Migration			
**CCl6**	Chemokine signaling pathway; Cytokine-cytokine receptor interaction	**Actn1**	Tight junction; Regulation of actin cytoskeleton; Focal adhesion; Adherens junction; Leukocyte transendothelial migration; Amoebiasis
**Cd5l**	inflammatory response, lipid synthesis, migration	**Akt1**	Fc gamma R-mediated phagocytosis; B cell receptor signaling pathway; Chemokine signaling pathway; Toll-like receptor signaling pathway; Focal adhesion; Tight junction; T cell receptor signaling pathway; Ras signaling pathway; mTOR signaling pathway; Jak-STAT signaling pathway
**Cxcr4**	Axon guidance; Cytokine-cytokine receptor interaction; Intestinal immune network for IgA production; Chemokine signaling pathway; Leukocyte transendothelial migration	**Cadm1**	Neural system cell adhesion
**Glg1**	Cell adhesion molecules (CAMs)	**Col6a2**	ECM-receptor interaction; Focal adhesion; PI3K-Akt signaling pathway
**Grb2**	Ras signaling pathway; Focal adhesion; B cell receptor signaling pathway; Gap junction; Chemokine signaling pathway; Natural killer cell mediated cytotoxicity; PI3K-Akt signaling pathway; MAPK signaling pathway	**Ctnna1**	Tight junction; Leukocyte transendothelial migration; Adherens junction

**Il16**	cytokine signaling pathway, unclassified to CD4	**Ctnnb1**	Leukocyte transendothelial migration; Focal adhesion; Tight junction; Adherens junction
**Itga2b**	Platelet activation; Hematopoietic cell lineage; Rap1 signaling pathway; Focal adhesion; PI3K-Akt signaling pathway; Regulation of actin cytoskeleton; phagosome	**Elmo1**	Chemokine signaling pathway; Rac protein signal transduction
**Itgb3**	Focal adhesion; Rap1 signaling pathway; Hematopoietic cell lineage; Platelet activation; ECM-receptor interaction; Regulation of actin cytoskeleton; Phagosome; PI3K-Akt signaling pathway; phagosome	**Epb4.1**	Tight junction
**Tmsb4x**	Regulation of actin cytoskeleton	**Itga5**	Regulation of actin cytoskeleton; Phagosome; Focal adhesion
**Pik3r1**	Chemokine signaling pathway; Toll-like receptor signaling pathway; Natural killer cell mediated cytotoxicity; Fc gamma R-mediated phagocytosis; Fc epsilon RI signaling pathway; B cell receptor signaling pathway; PI3K-Akt signaling pathway; Inflammatory mediator regulation of TRP channels; Regulation of actin cytoskeleton; Leukocyte transendothelial migration; TNF signaling pathway; Axon guidance; T cell receptor signaling pathway; Ras signaling pathway	**MyD88**	Toll-like receptor signaling pathway; NF-kappa B signaling pathway; MAPK signaling pathway
**Cmklr1**	chemotaxis, complement receptor mediated signaling pathway, immune response, inflammatory response	**Mylk**	Calcium signaling pathway; Regulation of actin cytoskeleton; Focal adhesion
		**Ncf2**	Leukocyte transendothelial migration; Phagosome
		**Parvb**	Focal adhesion
		**Pik3r5**	Ras signaling pathway; Regulation of actin cytoskeleton; Chemokine signaling pathway; Natural killer cell mediated cytotoxicity; Leukocyte transendothelial migration; Inflammatory mediator regulation of TRP channels; Focal adhesion
		**Plcg2**	Natural killer cell mediated cytotoxicity; Ras signaling pathway; NF-kappa B signaling pathway; Inflammatory mediator regulation of TRP channels; Axon guidance; Leukocyte transendothelial migration; B cell receptor signaling pathway
		**Ppp1cc**	Focal adhesion; Inflammatory mediator regulation of TRP channels; Long-term potentiation; Regulation of actin cytoskeleton
		**Prkcb**	gap junctions
		**Pten**	Tight junction; Focal adhesion; regulation of ERK1 and 2 cascade

		**Ptk2b**	chemokine signaling pathway, positive regulation of ERK1 and 2 cascade
		**Rasa1**	Axon guidance; MAPK signaling pathway; Ras signaling pathway
		**Tgfbr1**	Adherens junction; TGF-beta signaling pathway; MAPK signaling pathway; Cytokine-cytokine receptor interaction
		**Was**	Fc gamma R-mediated phagocytosis; Chemokine signaling pathway; Regulation of actin cytoskeleton; Adherens junction
	D) Cell adhesion		
**CD22**	Cell adhesion molecules (CAMs); Hematopoietic cell lineage; B cell receptor signaling pathway	**Arpc1b**	actin polymerization, regulation of actin cytoskeleton
**H2-Aa**	Antigen processing and presentation; Th1 and Th2 cell differentiation; Phagosome; Cell adhesion molecules (CAMs), MHCII; hematopoiesis	**Braf**	Regulation of actin cytoskeleton; Chemokine signaling pathway; Focal adhesion; Long-term depression; Natural killer cell mediated cytotoxicity; Endocrine resistance; Long-term potentiation
**H2-Ab1**	Phagosome; Th1 and Th2 cell differentiation; Cell adhesion molecules (CAMs); Antigen processing and presentation; MHCII	**Myl6**	regulation of actin cytoskeleton
**Icam1**	Cell adhesion molecules (CAMs); NF-kappa B signaling pathway; Natural killer cell mediated cytotoxicity;; Leukocyte transendothelial migration; macrophages T-cell adhesion and T cell to target cell		
**Parvg**	focal adhesion		
**Shc1**	Natural killer cell mediated cytotoxicity; Chemokine signaling pathway; Focal adhesion; Ras signaling pathway; chemokine signaling pathway		
**Stmn1**	MAPK signaling pathway		
**Thbs1**	Focal adhesion; ECM-receptor interaction; Phagosome; PI3K-Akt signaling pathway; Phagosome		
	E) Apoptosis		
**Casp8**	apoptosis	**Aifm2**	necroptosis
**Ct(bs,sa,sb, sc, sd, sf, sg, sl, ss, sz)**	Cathepsine, Lysosomal, Apoptosis	**Capn1**	Apoptosis
**Fadd**	Apoptosis; TNF signaling pathway; Toll-like receptor signaling pathway	**Chuk**	survival factor, (IKK), apoptosis
**Gadd45**	Apoptosis	**Traf2**	apoptosis, necroptosis
**Glul**	necroptosis		
**Mcl1**	Jak-STAT signaling pathway; Apoptosis; PI3K-Akt signaling pathway		

**Ppid**	necroptosis		
**Rbck1**	NOD-like receptor signaling pathway, necroptosis		
**Spna2**	apoptosis		
**Vdac1**	Calcium signaling pathway;; necroptosis		
F) Hematopoiesis			
**Cd38**	Hematopoietic cell lineage	**Cdk6**	PI3K-Akt signaling pathway; Cell cycle; hematopoiesis
**Csf1**	Hematopoietic cell lineage; TNF signaling pathway; Ras signaling pathway; Cytokine-cytokine receptor interaction; PI3K-Akt signaling pathway		
**Gp1bb**	hematopoiesis		
G) Inflammation			
**Adcy3**	Rap1 signaling pathway; Chemokine signaling pathway; Inflammatory mediator regulation of TRP channels; Longevity regulating pathway;	**Acsl5**	Peroxisome; PPAR signaling pathway
**Hgs**	Endocytosis; Phagosome	**Bcl2l1**	Jak-STAT signaling pathway; Chronic myeloid leukemia; Ras signaling pathway; PI3K-Akt signaling pathway; NF-kappa B signaling pathway
**Mpo**	Transcriptional misregulation in cancer; Phagosome	**Bmp2k**	cytokine signaling pathway
		**Coro2a**	Phagosome
		**Pla2g4a**	MAPK signaling pathway; Fc gamma R-mediated phagocytosis; Ras signaling pathway; Long-term depression; Inflammatory mediator regulation of TRP channels
		**Prkch**	Tight junction; Inflammatory mediator regulation of TRP channels;
		**Ptpn7**	MAPK signaling pathway
		**Rab5a**	Amoebiasis; Ras signaling pathway; Phagosome;
		**Trpv2**	Inflammatory mediator regulation of TRP channels; NOD-like receptor signaling pathway
		**Vamp3**	SNARE interactions in vesicular transport; Phagosome
H) Lysosome			
**Aga**	Lysosome; Other glycan degradation	**Ctns**	Lysosome
**Dnase2a**	Lysosome	**Hgsnat**	Glycosaminoglycan degradation; Lysosome
**Gaa**	Lysosome	**Scarb1**	Lysosome and phagosome
**Galns**	Glycosaminoglycan degradation; Lysosome		
**Gba**	Lysosome; Other glycan degradation		

**Gga1**	Lysosome		
**Gm2a**	Lysosome		
**Gns**	Metabolic pathways; Lysosome		
**Idua**	Lysosome		
**Napsa**	Lysosome		
**Npc2**	Lysosome		
**Pla2g15**	Lysosome; Glycerophospholipid metabolism		
I) Other			
**Cox7a2**	Oxidative phosphorylation	**Dapk3**	Autophagy, ER stress
**Map4k1**	MAPK signaling pathway	**Pik3c3**	Autophagy; Phagosome; Metabolic pathways
**Ndufa2**	Oxidative phosphorylation	**Prkcd**	Autophagy
**Ndufv**	oxidative phosphorylation	**Rps6ka1**	Thermogenesis
**Uqcrfs1**	Metabolic pathways; Oxidative phosphorylation	**Rptor**	Thermogenesis
		**Stx5a**	Autophagy

Supplementary Table 2. Overview of regulated proteins in untreated G2A-deficient and wild-type BMDMs. Description according to KEGG pathway description.

Downregulated		Upregulated	
		**A) Immune system processes**	
**Sos1**	Regulation of actin cytoskeleton; Gap junction; T cell receptor signaling pathway; Chemokine signaling pathway; Fc epsilon RI signaling pathway; Focal adhesion; B cell receptor signaling pathway; Ras signaling pathway; PI3K-Akt signaling pathway; Natural killer cell mediated cytotoxicity	**C1qa**	Complement and coagulation cascades
**Tradd**	NF-kappa B signaling pathway; TNF signaling pathway	**Plaur**	Complement and coagulation cascades
		**Serpinb2**	Complement and coagulation cascades
		B) Lipid metabolic processes	
		**Gpx7**	Glutathione metabolism; Arachidonic acid metabolism
		C) Migration	
**Dock2**	Chemokine signaling pathway; Fc gamma R-mediated phagocytosis	**Col6a2**	ECM-receptor interaction; Focal adhesion; PI3K-Akt signaling pathway
**Elmo1**	Chemokine signaling pathway	**Epb4.1**	tight junctions
**Irak4**	Toll-like receptor signaling pathway, cytokine signaling pathway	**Flnb**	MAPK signaling pathway; Focal adhesion
**Map2k2**	MAPK signaling pathway; Toll-like receptor signaling pathway; Natural killer cell mediated cytotoxicity; Long-term potentiation; Regulation of actin cytoskeleton; B cell receptor signaling pathway; T cell receptor signaling pathway; Ras signaling pathway; Gap junction	**Hspb1**	Amoebiasis; MAPK signaling pathway;
**Pik3r1**	Chemokine signaling pathway; Toll-like receptor signaling pathway; Natural killer cell mediated cytotoxicity; Inflammatory mediator regulation of TRP channels; Focal adhesion; Regulation of actin cytoskeleton; Leukocyte transendothelial migration; TNF signaling pathway; Axon guidance; Ras signaling pathway	**Mylk**	Calcium signaling pathway; Regulation of actin cytoskeleton; Focal adhesion
**Was**	Fc gamma R-mediated phagocytosis; Chemokine signaling pathway; Regulation of actin cytoskeleton; Adherens junction	**Prkca**	Leukocyte transendothelial migration; Ras signaling pathway; Fc gamma R-mediated phagocytosis; Gap junction; Natural killer cell mediated cytotoxicity; Long-term potentiation; MAPK signaling pathway; Inflammatory mediator regulation of TRP channels; Axon guidance; Calcium signaling pathway; Tight junction

		**Serpine1**	Complement and coagulation cascades
		**Tgfbr1**	Adherens junction; TGF-beta signaling pathway; MAPK signaling pathway; Cytokine-cytokine receptor interaction
	D) Cell adhesion		
		**H2-Aa**	Antigen processing and presentation; Th1 and Th2 cell differentiation; Phagosome; Cell adhesion molecules (CAMs), MHCII; hematopoiesis
		**Thbs1**	Focal adhesion; ECM-receptor interaction; Phagosome; PI3K-Akt signaling pathway; Phagosome
	E) Apoptosis		
**Bcl2l1**	Jak-STAT signaling pathway; Chronic myeloid leukemia; Ras signaling pathway; PI3K-Akt signaling pathway; NF-kappa B signaling pathway		
	F) Hematopoiesis		
**Cdk6**	PI3K-Akt signaling pathway; Cell cycle; hematopoiesis		
	G) Inflammation		
**Casp1**	NOD-like receptor signaling pathway; Cytosolic DNA-sensing pathway; Pertussis	**Ltbp1**	TGF-beta signaling pathway
**Nfkb1**	Ras signaling pathway; Toll-like receptor signaling pathway; MAPK signaling pathway; B cell receptor signaling pathway; Chemokine signaling pathway; Th1 and Th2 cell differentiation; T cell receptor signaling pathway; NF-kappa B signaling pathway; TNF signaling pathway; PI3K-Akt signaling pathway	**Ube2i**	NF-kappa B signaling pathway
**Prkch**	Tight junction; Inflammatory mediator regulation of TRP channels		
**Rps6ka5**	TNF signaling pathway; MAPK signaling pathway		
	H) Other		
**Atp6v1 (a, b2, e1)**	Oxidative phosphorylation; Metabolic pathways; mTOR signaling pathway; Synaptic vesicle cycle; Phagosome	**Ndufab1**	Oxidative phosphorylation
**Map4k1**	MAPK signaling pathway	**Rps6-ps4; Rps6**	HIF-1 signaling pathway; PI3K-Akt signaling pathway; Insulin signaling pathway
**Ndufa11**	Oxidative phosphorylation		
**Prkacb**	MAPK signaling pathway		
**Sdhb**	Oxidative phosphorylation		
